# Relationship between social cohesion and the care burden of primary family caregivers in central Tokyo, Japan

**DOI:** 10.1002/hsr2.238

**Published:** 2021-02-02

**Authors:** Yuki Naganuma, Kazue Yamaoka, Kenzo Takahashi

**Affiliations:** ^1^ Teikyo University Graduate School of Public Health Tokyo Japan

**Keywords:** family caregivers, home visiting care, J‐ZBI_8, social cohesion, social support, Zarit Care Burden Interview Scale

## Abstract

**Objectives:**

To clarify how the care burden of primary family caregivers is associated with social cohesion in an urban area of Tokyo, Japan.

**Study design:**

Cross‐sectional study.

**Methods:**

A questionnaire survey of primary family caregivers was conducted in Tokyo in 2015. Social cohesion was examined using the social capital indicators of Kondo et al, and the care burden of primary family caregivers was assessed by the Zarit Care Burden Interview Scale in Japanese short version (J‐ZBI_8). Data were analyzed by multiple regression models. Ethics approval was obtained to carry out this research.

**Results:**

Seventy‐nine caregivers responded to the questionnaires. After excluding 6 caregivers who did not respond appropriately to the questionnaire, 73 caregivers were included in the analyses. The average age of the primary family caregivers was 68.9 ± 12.7 years old, and that of the patients receiving care was 83.1 ± 10.0 years old. “Receipt of instrumental support” was significantly associated with reduced burden of care for family caregivers as assessed by the J‐ZBI_8 score (*P* = .027).

**Conclusion:**

This study suggested that social cohesion was significantly associated with reduced burden of care for primary family caregivers. Especially, the results suggested that “social support: receipt of instrumental support” was associated with a lower burden of care after adjustment for confounding factors. It is important to understand family structure and social community differences such as informal social support for future policy making.

## 1 INTRODUCTION

Serious concerns have been raised about family caregiving of older adults in many countries. There is not only a growing demand for families to provide elderly care but also growing evidence that caregiving itself poses risks related to mental, physical, and economic health for some people. In line with the increasing elderly population in Japan,^1^ the Ministry of Health, Labour and Welfare aims to maintain the dignity of the elderly and support independent living so that they can live their own lives as much as possible in the community.^2^ It is essential to provide comprehensive and continuous home care in cooperation with local medical and nursing care agencies. However, as life expectancy lengthens and age and family structure change, concerns are emerging about both the lack and burden of elderly care.^3^ Zarit et al^4^ define care burden as “the degree of damage the caregiver suffers in terms of emotional, physical health, social life and financial condition as a result of caring for a relative.”

Long‐term care insurance systems provided in Japan to support elderly care currently classify patients based on their activities of daily life or level of dementia. At present, the type of family structure in which a patient lives is not considered a criterion for registration. Family composition is greatly influenced by the promotion of home care. For elderly people who are admitted to hospital, most factors making it difficult to discharge them from hospital are related to nursing care issues.^5^, ^6^ The availability of care tends to be affected by the situation of the caregivers. Regional long‐term care systems never work without the support of the local community.

Some studies show that support from the community depends on its social cohesion. Nevertheless, there is little evidence on whether social cohesion reduces the burden of caregivers who provide home nursing care. Social support is believed to promote health by reducing mental stress.^7^ Chan et al^8^ define social cohesion as a state of affairs concerning both the vertical and the horizontal interactions among members of society as characterized by a set of attitudes and norms that include trust, a sense of belonging, and the willingness to participate and help, as well as their behavioral manifestations. Cramm et al^9^ found that neighborhood social capital and social cohesion are accessed via individual, group, or community membership, and these resources are norms of reciprocity, civic participation, trust in others, and benefits of membership. Putnam^10^ stated that social capital refers to physical objects, and human capital refers to the properties of connections among individuals, social networks, and the norms of reciprocity and trustworthiness that arise from them. Social support is broadly defined as both emotional and instrumental support. Noguchi^11^ said that emotional support is support that works on emotions, such as “listening to worries and worries” and “enhancing,” and instrumental support is “nursing and care” and “lending money.” Social support is a means of substantive support.

In a study in the United States on the association of social capital with depression, the risk of depression was reduced by 57% in a group of local residents with high confidence compared to the group with low confidence.^12^ In a survey conducted in Japan that examined regional differences in the burden of care for the family caring for their elderly showed that there was better nursing care and a higher care burden in urban areas than in sparsely populated areas.^13^ However, after correcting for the factors related to care burden, the tendency for higher care burden in urban areas was no longer remarkable. The relationship between social capital and depression has been well discussed,^7^, ^12^, ^13^, ^14^ and numerous papers have reviewed the care burden of primary caregivers.^15^ There is also research on regional differences in care burden.^16^ However, the relationship between the care burden of home caregivers and regional social cohesion has been little explored. The present study aimed to clarify the relationship between social cohesion and family care burden to support the development of an enhanced caregiver support system by use of a questionnaire survey. We also discuss the difference between two areas, namely the center of Tokyo (this study) and a rural island (a previous study conducted using the same questionnaire)^17^ in Japan.

## 2 METHODS

### 2.1 Subjects

The survey was conducted as a placement method survey using a self‐administered questionnaire. The study subjects were primary family caregivers who lived with patients over the age of 65 who contracted with visiting nurse services in Tokyo. The home‐visit nursing system in Japan is based on medical insurance and long‐term care insurance. Funding is provided through health insurance premiums paid by the insured, contributions from working‐age individuals, and public sources. When medical treatment is received, the co‐payment is 20% (30% for individuals with incomes over a certain level). When visiting nursing services are used, the co‐payment rate is 10% (or 20% or 30%). In 2015, 796 home‐visiting nurse offices were operating in Tokyo. With cooperation from five visiting nursing offices, each located in an adjacent ward in Tokyo, a questionnaire survey was conducted during home visits to service recipients. The average number of patients requiring long‐term care during the survey at the five visiting nursing establishments was 261 patients for 3 months. As this study was intended for the primary caregivers, elderly long‐term care insurance recipients living alone were excluded from the survey. The 261 patients were the average number of patients over the implementation period of 3 months and include only patients classified as long‐term care level 1 to 5. Hospitalized patients were excluded. Those who did not sign the consent form and those who did not respond to the Japanese short version of the Zarit Care Burden Interview Scale (J‐ZBI_8) were also excluded from the analysis. The questionnaire was distributed to the family caregiver by the home‐visiting nurse, and the family caregiver filled out the questionnaire, which was then collected by the home‐visiting nurses. The survey was conducted from October 9 to December 9, 2015.

### 2.2 Questionnaires

The questionnaire contents are explained in detail in a previous study conducted in an island area of Japan.^17^ The questionnaire assessed the following categories.

Social capital indicators: The questionnaire for social capital indicators of Kondo et al^18^ was used, and the questions regarding social capital that were investigated are as follows:

- Social support: receipt of emotional support for the caregiver by the community (Yes/No).
- Social support: emotional support provided to others by the caregiver (Yes/No).
- Social support: instrumental support provided; care for the resident given by others when the caregiver cannot provide care (Yes/No).
- Social support: instrumental support provided by the caregiver and others (Yes/No).
- Participant in organized activities: frequency of participation in the activities of a group organization.
- Caregiver's social network: using the frequency of meeting with friends and acquaintances as a question item.

Questions e and f asked for frequency, but we classified them into two (Yes/No) categories. The J‐ZBI_8^19^ was also used. The rating scale of the J‐ZBI_8 (with scores ranging from 0 to 32) is an objective measure that can be used as an international comparison to capture the care burden of caregivers.^4^, ^20^ The short version is widely used.^21^, ^22^

Confounding factors included caregiver's sex, age, and occupation, length of caregiving, educational background, length of residence time, household, sex of the patient under care, and patient's age.

### 2.3 Statistical analyses

To confirm internal consistency, Cronbach's *α* ^19^ was obtained for the J‐ZBI_8. Characteristics of the participants are summarized as the mean with SD or median (Q1, Q3) for continuous variables and number (%) for categorical variables. The score of J‐ZBI_8 was used as an outcome value after log‐transformation, and the effects of the exploratory variables were analyzed using simple and multiple regression models. Social capital variables of support variables a, b, c, and d and participation in organized activities (e) and social networks (f) were used along with the other confounding factors.

Arai and Zarit^23^ used receiver operating characteristic analysis of the J‐ZBI_8 to determine a reference cutoff value as an indicator of depressive symptoms. The total score of the J‐ZBI_8 was classified into a dichotomous variable of ≥13 vs ≤12, and a logistic regression model was used with the odds ratio and associated 95% confidence interval (CI) calculated.

In the multiple regression analyses, a stepwise method (with inclusion and exclusion criteria selected at the 20% significance level) was used as a method to select variables. A two‐sided significance level of 5% was considered to indicate statistical significance. All statistical analyses were performed using SAS Ver. 9.4.

### 2.4 Ethical considerations

This study was carried out in accordance with the Declaration of Helsinki, a set of ethical principles regarding human experimentation for the medical community. To protect personal information, all information remained anonymous. Informed consent of each subject to participate in this study was obtained by their signing of the questionnaire document. This study was aimed at the primary caregiver who is a member of the patient's family, and the only patient information collected was sex and age. Only the primary caregivers signed the questionnaire. Ethics approval was granted by the ethics committee of Teikyo University (approval no. 15‐038) and that of the Tokyo‐Hokuto Health Co‐operative Association (approval no. 76).

## 3 RESULTS

Among the primary caregivers surveyed, 79 responded (the number of patients serviced by the five home‐visit nursing stations, including the elderly living alone, was 261. Responses were obtained from 79 primary caregivers of these patients, giving a response rate of 30.3%). After we excluded six caregivers who did not respond to the questionnaire, 73 primary family caregivers were included in the analysis. Because this survey was intended for the primary caregivers, the number of patients included those living alone. Therefore, the exact response rate was expected to be higher than 30%. The average age of the primary family caregivers was 68.9 ± 12.7 years old, and they included 23 men (31.5%) and 50 women (68.5%). The average age of the patients receiving care was 83.1 ± 10.0 years old, and they included 31 men (43.7%) and 40 women (56.3%) (Table 1). Cronbach's *α* for the J‐ZBI_8 was .89.

**TABLE 1:** Characteristic of the participants (*N* = 73)

	Total	Missing	Number	%	Mean (SD)	Median (25th‐75th percentile)
Social capital scale	66	7			4.3 (1.5)	5.0 (4.0‐5.0)
a: Social support: receipt of emotional support	72	1				
No			7	9.7		
Yes			65	90.3		
b: Social support: emotional support provided	71	2				
No			12	16.9		
Yes			59	83.1		
c: Social support: receipt of instrumental support	72	1				
No			18	25.0		
Yes			54	75.0		
d: Social support: instrumental support provided	67	6				
No			21	31.3		
Yes			46	68.7		
e: Participant in organized activities	72	1				
No			40	55.6		
Yes			32	44.4		
f: Caregiver's social network	72	1				
No			19	26.4		
Yes			53	73.6		
Caregiver						
Gender						
Male	73	‐	23	31.5		
Female			50	68.5		
Age (years)	72	1			68.9 (12.7)	71.0 (62.0‐78.5)
Length of residence time	70	3			36.6 (18.7)	40.0 (23.0‐50.0)
Length of caregiving time	71	2			6.3 (6.7)	4.1 (2.0‐8.0)
Family members present (excepting care recipient)						
Have	72	1	33	45.8		
Not have			39	54.2		
Working status						
Working^a^	68	5	46	67		
Unemployed/homemaker			22	32.4		
Education						
High school	66	7	13	19.7		
Graduated high school			28	42.4		
Higher than high school			25	37.9		
Home‐care patient						
Gender						
Male	71	2	31	43.7		
Female			40	56.3		
Age					83.1 (10.0)	85.0 (76.0‐90.0)
Pattern of care						
Elder to elder	71	2	37	52.1		
Next‐generation primary caregivers^b^			34	47.9		
Gender pattern						
Male‐Male	71	2	4	5.6		
Male‐Female			18	25.4		
Female‐Male			27	38.0		
Female‐Female			22	31.0		

Abbreviation: CI, confidence interval; SD, standard deviation.

^a^ Part‐time work is included.

^b^ Includes daughter, daughter‐in‐law, son, and grandchild.

The results of the single and multiple regression analyses are shown in Table 2. After adjustment for confounding factors, the results of the regression model with stepwise variable selection showed a significant difference for “social support: receipt of instrumental support” (*P* = .027). The results of the logistic regression analysis are shown in Table 3. Similar to the results of the multiple regression analysis, the odds ratio of having or not having “social support: receipt of instrumental support” was 0.24 (95% CI = 0.07‐0.81, *P* = .024). The results of both analyses indicated that the receipt of instrumental support was associated with less care burden after adjustment for confounding factors.

**TABLE 2:** Results of linear regression analysis

	Simple regression analysis			Multiple regression analysis		
	*β* (beta)	SE	*P* value	*β* (beta)	SE	*P* value
Social capital total^a^	−.05	0.03	.115	‐		
a: Social support: receipt of emotional support	−.09	0.16	.577	‐		
b: Social support: emotional support provided	−.16	0.12	.188	‐		
c: Social support: receipt of instrumental support	−.23	0.10	.029	−.24	0.11	.027
d: Social support: instrumental support provided	−.14	0.10	.144	‐		
e: Participant in organized activities	−.07	0.09	.426	‐		
f: Caregiver's social network	−.04	0.11	.686	‐		
Caregiver						
Gender	.08	0.10	.403	‐		
Age (10‐year units)	−.04	0.04	.303	‐		
Length of residence time (1‐year units)	−.05	0.02	.094	‐		
Length of caregiving time (1‐year units)	.02	0.08	.805	‐		
Family members present (excepting care recipient)	.05	0.05	.278	‐		
Working status	−.01	0.10	.909	‐		
Education						
High school	−.11	0.12	.369	‐		
Graduated high school	−.07	0.09	.465	‐		
Higher than high school	.18	0.09	.059	.18	0.09	.054
Home‐care patient						
Gender	.02	0.09	.820	‐		
Age (10‐year units)	.01	0.05	.763	‐		
Gender pattern						
Age	−.04	0.09	.648	‐		
Gender						
Male‐Male	−.08	0.19	.693	‐		
Male‐Female	.00	0.11	.988	‐		
Female‐Male	−.01	0.09	.925	‐		
Female‐Female	.02	0.10	.869	.05	0.10	.5964

^a^ Not included in the linear regression analysis. A stepwise method was used for variable selection in the multiple linear regression analysis with the inclusion and exclusion criteria of 20%. Variables not selected in the stepwise method are indicated by “‐”.

**TABLE 3:** Results of logistic regression analysis

	Simple regression analysis				Multiple regression analysis			
	OR	95% CI		*P* value	OR	95% CI		*P* value
Social capital total^a^	0.74	0.51	1.04	.089				
a: Social support: receipt of emotional support	0.54	0.09	3.11	.468	‐			
b: Social support: emotional support provided	0.42	0.11	1.55	.190	‐			
c: Social support: receipt of instrumental support	0.34	0.10	1.05	.063	0.24	0.07	0.81	.024
d: Social support: instrumental support provided	0.63	0.21	1.88	.405	‐			
e: Participant in organized activities	0.56	0.20	1.52	.263	‐			
f: Caregiver's social network	0.55	0.18	1.69	.288	‐			
Caregiver								
Gender	1.16	0.40	3.55	.786	‐			
Age (10‐year units)	0.92	0.62	1.38	.698	‐			
Length of residence time (1‐year units)	0.77	0.54	1.06	.112	‐			
Length of caregiving time (1‐year units)	1.01	0.39	2.44	.974	‐			
Family members present (excepting care recipient)	1.03	0.25	3.84	.961	1.68	0.95	3.08	.081
Working status	0.75	0.24	2.18	.604	‐			
Education								
High school	1.03	0.25	3.84	.961	‐			
Graduated high school	0.49	0.16	1.36	.179	‐			
Higher than high school	1.74	0.63	4.82	.283	‐			
Home‐care patient								
Gender	0.76	0.28	2.07	.590	‐			
Age (10‐year units)	0.97	0.59	1.63	.916	‐			
Gender pattern								
Age	1.18	0.44	3.24	.743	‐			
Gender								
Male‐Male	0.58	0.03	4.85	.649	‐			
Male‐Female	1.11	0.33	3.46	.865	‐			
Female‐Male	1.42	0.52	3.88	.488	‐			
Female‐Female	0.63	0.20	1.85	.416	‐			

Abbreviations: CI, confidence interval; OR, odds ratio.

^a^ Not included in the logistic regression analysis. A stepwise method was used for variable selection in the multiple logistic regression analysis with the inclusion and exclusion criteria of 20%. Variables not selected in the stepwise method are indicated by “‐”.

## 4 DISCUSSION

The relationship between a caregiver's social cohesion and primary family care burden was examined via a placement method survey in the Tokyo metropolitan area. The results suggested that “social support: receipt of instrumental support” was associated with less care burden after adjustment for confounding factors.

Among the items in this survey referring to social support, “instrumental support provided care for the resident given by others when the caregiver cannot provide care” tended to increase the care burden of the home caregiver. The primary caregiver may have anxiety about his/her health condition, and when it deteriorates, there may be no one the caregiver can rely on, and thus their burden of care may potentially increase. Similar results were observed in the island survey, that is, there was a significant difference in the results related to “social support.”^17^ In addition, there were significant differences in social support related to “reception of emotional support” and “participation in organized activities” in the island survey (Appendix Table A1, A2).

The primary family caregivers who live on islands tended to seek emotional support and participation in organizational activities (social and positive self‐role). In contrast, among those living in Tokyo, the burden of care for the primary caregivers was not associated with “emotional support” or “participation in organizational activities,” which indicates the frequency of participation in the activities of a group organization, but rather was associated with “having receipt of instrumental support”. Surveys in urban and rural areas in South Australia and Poland have shown that rural areas have a higher level of attachment to land, levels of networks, citizen participation, and social cohesion.^24^, ^25^ The social environment may be a possible factor contributing to the difference between urban and rural areas. Morelli et al^26^ pointed to unequal access to health and social care, and they said there is an urgent need for culturally savvy reflections on the anthropological and psychosocial characteristics of various rural communities to improve them. In the present study, 54.2% of the primary family caregivers in Tokyo responded that no family members other than primary caregivers and the recipient of care live together (Table 1). In comparison, only 30.8% of the island's primary family caregivers did not live with family other than the recipient of care. This result is affected by the difference in household structure and the average number of household members in each prefecture in Japan.^27^ The number of single households and nuclear family households tends to increase as people locate to the city centers in Japan.

In 2015, the percentages of employees who changed their jobs due to their requirement to provide home care were 0.6% for men and 1.1% for women.^28^ Even if they do not quit their jobs, there may be situations in which employment status must change. According to the Basic Employment Equality Survey, the ratio of businesses with a nursing care provider leaving the system in 2017 was 70.9% for those with five or more employees and 90.9% for those with 30 or more employees.

The nursing care leave system is not adequate in Japan. Only 2.0% of business establishments had employees who took nursing care leave between April 1, 2016, and March 31, 2017.^29^ According to the “Survey on Men and Women Employment Management in Companies in Response to the Revised Child Care and Care Leave Act,” the ratio of establishments with employees who had taken care leave in the past year was 9.7% based on paid hourly leave figures.^30^ Only a small percentage of employees had reduced their working hours. It may also be difficult for caregivers to keep working, even if they have reduced their working hours, and there may be situations in which work style must be changed to provide care.

In the long‐term care insurance system, the degree of care required is determined without taking either family background or family structure into consideration, so it is essential to consider the care burden of the primary caregiver. It has been suggested that caregivers receive partial support, especially in areas with weak social cohesion. For primary caregivers in central Tokyo, “participation in organizational activities” did not affect the level of care burden, possibly because no family member other than the caregiver is available to provide elderly care and also to go to work to earn money. According to the results of this survey, the less social support available as “instrumental support”, the higher is the burden of long‐term care. There is no one to care for the patient other than the main caregiver, who must take care of the patient every day and thinks that he/she must provide this care alone. Considering the Japanese family structure, it can be said that there are many hurdles to promoting home care.

Home caregivers and primary family caregivers who are unable to “participate in organizational activities” are required to further strengthen their social ties when seeking support from local community members and social support from medical social workers when their care recipient returns from the hospital to the community. In medical institutions, medical social workers and nurses mainly support the patient's discharge. However, as the length of hospital stay is being reduced, the problems faced by patients are becoming more complex, and increasing social support is becoming a challenge.^31^

Recently, United Kingdom introduced Social Prescribing, which allows third parties (called link workers) to connect to local resources. One report found that Social Prescribing saved £647 000 (about 100 million Japanese yen).^32^ Kumakawa et al^33^ pointed out that various organizational activities and regional resource information of organizations are not systematically organized. In addition, they suggested that informal regional resources need to be identified and developed. Informal social resources will become increasingly necessary not only for caregivers but also for the patients and local residents.

Kawashima^34^ noted that it is important for social workers to systemize informal services such as local volunteers, which are components of social capital. For better mobilization of social capital including local volunteers, social welfare councils, and community care centers that work in the service of community people should actively grasp the level of available local resource information including human resources. The goal is to establish a community where local residents and various local players participate beyond a relationship between supporters and receivers. In the future, there will be challenges in understanding and operating community support coordination and community resources for informal care in Japan.

### 4.1 Limitations

The present study showed the importance of community cohesion in possibly reducing the burden on home caregivers. The strength of this research is that the interviews with caregivers providing elderly care were successfully conducted by home‐visiting nurses. However, this study does have some limitations. The type and frequency of social security services used by home caregivers could not be included in the question items. Therefore, it is not possible to adjust for the sense of care burden that may be affected by the receipt of social security services.

Moreover, as the only available information on the background of the caregiver was sex and age, adjustments were required for activities of daily living, daily living independence, cognitive symptoms, and disease. According to Sugiura et al,^35^ the care burden of the caregiver is affected by the patient's cognitive impairment. Similarly, in a survey conducted by Yu et al^36^ in those with Alzheimer's disease and their caregivers in China, cognitive level and caregiving were directly related to caregiver burden. Social support, family functioning, and care experience can mediate the relationship between patient factors and caregiver burden. These factors could not be analyzed as adjustment factors, so it may be possible that the sense of care burden was affected in caregivers caring for recipients with significant cognitive impairment or behavioral and psychological symptoms of dementia.

The care level of long‐term care insurance could not be adjusted for in this study. However, Kuwahara et al^37^ reported that caregivers with heavy care burdens have a higher proportion of chronic illnesses than those with light care burdens, those with more time to provide physical care and observation, and those using more types of home services. As the physical condition of the primary caregiver is greatly influenced by the sense of care burden, it may be necessary to consider adjusting the level of care required.

Because the number of variables was large and the number of survey subjects was small, it was difficult to use certain items as adjustment factors, and thus the degree of care required could not be adjusted as a variable in the analysis. Furthermore, it was not possible to identify all of the elderly living alone. In addition, some patients receiving care were admitted to or discharged from the hospital during the survey period, and thus an exact response rate could not be calculated.

A recent study found that social support for caregivers is associated with physical effects and psychological stress.^38^ This suggests the possibility that, to reduce care burden, interventions should focus more on “feeling connected” rather than on “building connections.”

## 5 CONCLUSION

Despite the above limitations, this study suggested that social cohesion was significantly associated with reduced burden of care for primary family caregivers who live in Tokyo. Especially, the results suggested that “social support: receipt of instrumental support” was associated with less care burden after adjustment for confounding factors. However, the type of support differed somewhat from that received by caregivers living in the island community we surveyed before. It is important to understand family structure and social community differences such as informal social support for future policy making.

## CONFLICT OF INTEREST

The authors declare no conflict of interest.

## AUTHOR CONTRIBUTIONS

Conceptualization: Yuki Naganuma, Kenzo Takahashi

Data Curation: Yuki Naganuma

Formal Analysis: Yuki Naganuma, Kazue Yamaoka

Investigation: Yuki Naganuma

Methodology: Yuki Naganuma, Kazue Yamaoka, Kenzo Takahashi

Project Administration: Yuki Naganuma

Supervision: Kazue Yamaoka, Kenzo Takahashi

Writing—Original Draft Preparation: Yuki Naganuma

Writing—Review & Editing: Yuki Naganuma, Kazue Yamaoka, Kenzo Takahashi

All authors have read and approved the final version of the manuscript.

Yuki Naganuma had full access to all of the data in this study and takes complete responsibility for the integrity of the data and the accuracy of the data analysis.

## TRANSPARENCY STATEMENT

The corresponding author (Y.N.) affirms that this manuscript is an honest, accurate, and transparent account of the study being reported; that no important aspects of the study have been omitted; that any discrepancies from the study as planned have been explained.

## Supporting information


**Appendix S1.** Supporting Information

## Data Availability

The datasets generated and/or analyzed during the current study are not publicly available due to ethical restrictions.

## Appendix

**TABLE A1:** Two‐area multiple regression analysis

	Multiple regression analysis (island)			Multiple regression analysis (Tokyo)		
	*β* (beta)	SE	*P* value	*β* (beta)	SE	*P* value
Social capital total^a^				‐		
a: Social support: receipt of emotional support	.49	0.18	.009	‐		
b: Social support: emotional support provided	‐			‐		
c: Social support: receipt of instrumental support	−.37	0.14	.010	−.24	0.11	.027
d: Social support: instrumental support provided	‐			‐		
e: Participant in organized activities	−.13	0.09	.171	‐		
f: Caregiver's social network	‐			‐		
Caregiver						
Gender	‐			‐		
Age (10‐year units)	‐			‐		
Length of residence time (1‐year units)	‐			‐		
Length of caregiving time (1‐year units)	‐			‐		
Family members present (excepting care recipient)	‐			‐		
Working status	‐			‐		
Education						
High school	‐			‐		
Graduated high school	‐			‐		
Higher than high school	‐			.18	0.09	.054
Home‐care patient						
Gender	‐			‐		
Age (10‐year units)	‐			‐		
Gender pattern						
Age	‐			‐		
Gender	−.42	0.22	.066			
Male‐Male				‐		
Male‐Female	‐			‐		
Female‐Male	‐			‐		
Female‐Female	‐			.05	0.10	.5964

Abbreviations: CI, confidence interval; OR, odds ratio.

^a^ Not included in the multiple regression analysis. A stepwise method was used for variable selection in the multiple regression analysis with the inclusion and exclusion criteria of 20%. Variables not selected in the stepwise method are indicated by “‐”.

**TABLE A2:** Two‐area logistic regression analysis

	Multiple regression analysis (island)			Multiple regression analysis (Tokyo)			
	OR	95% CI	*P* value	OR	95% CI		*P* value
Social capital total^a^							
a: Social support: receipt of emotional support	‐			‐			
b: Social support: emotional support provided	‐			‐			
c: Social support: receipt of instrumental support				0.24	0.07	0.81	.024
d: Social support: instrumental support provided	‐			‐			
e: Participant in organized activities	0.23	0.07‐0.66	.009	‐			
f: Care giver's social network	‐			‐			
Caregiver							
Gender	‐			‐			
Age (10‐year units)	‐			‐			
Length of residence time (1‐year units)	‐			‐			
Length of caregiving time (1‐year units)	‐			‐			
Family members present (excepting care recipient)	‐			1.68	0.95	3.08	.081
Working status				‐			
Education							
High school	‐			‐			
Graduated high school	‐			‐			
Higher than high school	‐			‐			
Home‐care patient							
Gender	0.74	0.71‐6.70	.189	‐			
Age (10‐year units)	‐			‐			
Gender pattern							
Age	‐			‐			
Gender							
Male‐Male	‐			‐			
Male‐Female	‐			‐			
Female‐Male	‐			‐			
Female‐Female	‐			‐			

Abbreviations: CI, confidence interval; OR, odds ratio.

^a^ Not included in the logistic regression analysis. A stepwise method was used for variable selection in the multiple logistic regression analysis with the inclusion and exclusion criteria of 20%. A variable that was not selected in the stepwise method is indicated by “‐”.
