# Supplementary material for: Relationship between social cohesion and the care burden of primary family caregivers in central Tokyo, Japan
Source: Health Sci Rep. 2021 Feb 2;4(1):e238. doi: 10.1002/hsr2.238 (PMC7853624; doi:10.1002/hsr2.238)
Supplement: Supplementary file 1 — Appendix S1. Supporting Information [file HSR2-4-e238-s001.pdf]

Questionnaire survey on community unity and family care burden

My name is Yuki Naganuma of the Graduate School of Public Health, Teikyo University. As a current research theme, we are investigating the relationship between community unity and the family care burden of home caregivers. The purpose contents of the survey are as follows. We would like to ask for your understanding of the purpose of the research and for your cooperation.

Kind regards

1, Research purpose

This is a questionnaire to investigate the relationship between the family care burden of the main caregiver who is providing care at home and the connection between the main caregiver's community.

2, Investigation method

We will conduct a simple questionnaire survey using a paper questionnaire. The main caregivers at home who are involved in home care are eligible, and the relevant items for home care are selected for their connection with society. The indicators used are the Zarit Care Burden Scale and Social Capital Indicators.

3. Free will and right to refuse to participate in the research

Participation and cooperation in this research is done of your own free will. In addition, even if you agree to participate or cooperate, you can withdraw your consent at any time without any disadvantage to you. Please do not hesitate to let us know if you are willing to participate in or cooperate with our research. Even if the person completing the questionnaire does not understand the contents of the questionnaire, etc., and responding is, if the intention to participate in the research can be confirmed, it is possible for this person to participate in the research by designating a consenting person. In that case, please have the consenting person sign this form and write the person's relationship and reason for consenting.

4. Expected benefits from research

It is possible to clarify how the burden of family care is changing by supporting social background and community unity, and how the locality reduces the burden on families involved in home care. Our goal is to clarify the informal support that are necessary to support home health care.

5. Privacy and handling of personal information

The information obtained in this survey will be input as anonymized data, and after the completion of the research, we will discard the collected data. We promise that the input data will not be used for anything other than for the purpose of the present research.

6. How the results of the research will be published
In addition to presentation of the results of this study at the Graduate School of Public Health, Teikyo University, the results may also be made public to related academic societies. We will give due consideration to the protection of personal information at the time of publication.

7. Contact information of the researcher

Name: Yuki Naganuma
Affiliation: Teikyo University, Graduate School of Public Health
Address: 173-8605 Kaga 2-11-1, Itabashi-ku, Tokyo
Tel.: 03-3964-3294 (ext. 46220 Reception, Graduate School of Public Health)
e-mail: sph-ynaganuma@med.teikyo-u.ac.jp:

If you understand the above description and can cooperate in completing this questionnaire, please sign the following.

Date
Name of the person or the consenting person

____

The questionnaire starts on the next page.

Question 1

Please answer these questions about you.

Age: ( ) years old
Sex: (Male, Female)
Occupation: (self-employed, private company, civil servant, teacher, part-time worker, student, unemployed, full-time housewife, etc.)
Provision of long-term care: (months)
Number of family members: ( )
Final education: (elementary and junior high school, high school, vocational school, various schools, technical college, junior college, university, graduate school, etc.)
Home ownership: (Yes or No)
Length of residence: (year month)

Question 2
Please tell us about the person you are taking care of.
Age: ( ) years old
Sex: (Male, Female)

Question 3
Who listens to your worries and complaints? Please choose the most applicable response from 1 to 7 with a circle.

1. Spouse
2. Child living together
3. Children or relatives living separately
4. Neighbors
5. Friends
6. Non-medical staff (food service, local fire brigade, community volunteers, etc.)
7. No one

Question 4

On the other hand, whose worries and complaints do you listen to? Please choose the most applicable response from 1 to 7 with a circle.

1. Spouse
2. Child living together
3. Children or relatives living separately
4. Neighbors
5. Friends
6. Non-medical staff (food service, local fire brigade, community volunteers, etc.)
7. No one

Question 5

Who will take care of patient if you must rest for a few days because of illness? Please choose the most applicable response from 1 to 7 with a circle.

1. Spouse
2. Child living together
3. Children or relatives living separately
4. Neighbor
5. Friends
6. Non-medical staff (food service, local fire brigade, community volunteers, etc.)
7. No one

Question 6

On the other hand, who will care for? Please choose the most applicable response from 1 to 7 with a circle.

1. Spouse
2. Child living together
3. Children or relatives living separately
4. Neighbor
5. Friends
6. Non-medical staff (food service, local fire brigade, community volunteers, etc.)
7. No one

Question 7

How often do you participate in meetings and groups such as the following: Political relations, industry groups, volunteer groups, clubs for the elderly, religion, sports, neighborhood associations, hobby groups?

Choose the most applicable from 1 to 6?

1. Almost every day
2. Two to three days a week
3. About once a month
4. Once or twice a month
5. Several times a year
6. I don’t participate

Question 8

How often do you meet friends and acquaintances? Choose the most applicable from 1 to 6.

1. Almost every day
2. Two to three days a week
3. About once a month
4. Once or twice a month
5. Several times a year
6. I don’t meet them.

Question 9

For each of the questions below, circle the number that you think applies to your feelings.

1. Do you think that you are troubled by the behavior of the person receiving care?
   0: I don’t think so 1: I rarely think so 2: I sometimes think so
   3: I frequently think so 4: I always think so

2. Do you get angry when you are near someone who is receiving care?
   0: I don’t think so 1: I rarely think so 2: I sometimes think so
   3: I frequently think so 4: I always think so

3. Do you think it's becoming harder to get along with family and friends because you have to provide nursing care?
   0: I don’t think so 1: I rarely think so 2: I sometimes think so

3: I frequently think so 4: I always think so

4. Do you think you can't rest when you're near someone who you are taking care for?
   0: I don’t think so 1: I rarely think so 2: I sometimes think so
   3: I frequently think so 4: I always think so

5. Do you think that your chances of participating in society have decreased because you have to provide nursing care?
   0: I don’t think so 1: I rarely think so 2: I sometimes think so
   3: I frequently think so 4: I always think so

6. Have you ever thought that you can't invite your friends because of the care of the patient at home?
   0: I don’t think so 1: I rarely think so 2: I sometimes think so
   3: I frequently think so 4: I always think so

7. Do you ever want to leave care to someone?
   0: I don’t think so 1: I rarely think so 2: I sometimes think so
   3: I frequently think so 4: I always think so

8. Do you think that you do not know what to do for the person who is receiving nursing care?
   0: I don’t think so 1: I rarely think so 2: I sometimes think so
   3: I frequently think so 4: I always think so

That’s all there is to this questionnaire. Thank you very much for your cooperation.
